# Use of Nuclear Spin Noise Spectroscopy to Monitor Slow Magnetization Buildup at Millikelvin Temperatures

**DOI:** 10.1002/cphc.201600323

**Published:** 2016-07-22

**Authors:** Maria Theresia Pöschko, David Peat, John Owers‐Bradley, Norbert Müller

**Affiliations:** ^1^ Institute of Organic ChemistryJohannes Kepler University LinzAltenbergerstrasse 694040LinzAustria; ^2^ School of Physics & AstronomyUniversity of NottinghamUniversity ParkNottinghamNG7 2RDUK; ^3^ 64 Canterbury roadPennWolverhamptonWV4 4EHUK; ^4^ Faculty of ScienceUniversity of South BohemiaBranišovská 1645/31A370 05České BudějoviceCzech Republic

**Keywords:** magnetic properties, nanoparticles, NMR spectroscopy, radiation damping, spin relaxation

## Abstract

At ultralow temperatures, longitudinal nuclear magnetic relaxation times become exceedingly long and spectral lines are very broad. These facts pose particular challenges for the measurement of NMR spectra and spin relaxation phenomena. Nuclear spin noise spectroscopy is used to monitor proton spin polarization buildup to thermal equilibrium of a mixture of glycerol, water, and copper oxide nanoparticles at 17.5 mK in a static magnetic field of 2.5 T. Relaxation times determined in such a way are essentially free from perturbations caused by excitation radiofrequency pulses, radiation damping, and insufficient excitation bandwidth. The experimental spin‐lattice relaxation times determined on resonance by saturation recovery with spin noise detection are consistently longer than those determined by using pulse excitation. These longer values are in better accordance with the expected field dependence trend than those obtained by on‐resonance experiments with pulsed excitation.

## 1 Introduction

High nuclear spin polarization levels can be attained by lowering the temperature to the millikelvin range. This effect has been exploited as an alternative means of generating nuclear hyperpolarization, even at room temperature by using the “brute‐force” approach.^1^, ^2^ It is critical for this approach to achieve high polarization levels within reasonably short time spans. Nuclear spin polarization buildup is a longitudinal relaxation process characterized by a relaxation time constant *T*_1_, which is “astronomically” slow (i.e. in the order of magnitude of 10^7^ s) below 1 K.^3^ Nanoparticles have been shown to have a huge effect in accelerating spin polarization buildup at very low temperatures.^4^, ^5^ These preceding studies demonstrated that the presence of metallic nanoparticles, for example, 30 nm platinum or copper, could reduce the *T*_1_ relaxation times by several orders of magnitude at ultralow temperatures. This finding opens up the possibility of using high magnetic field and low temperature to grow significant polarization in a reasonable timescale, and thereby, encourage the development of a brute‐force polarizer. The development of improved protocols for brute‐force polarization requires the slow buildup of large nuclear spin polarization at low temperatures to be monitored. There are several obstacles that need to be overcome to achieve this goal. One problem arises from strong radiation damping (RD) in the NMR circuit caused by large nuclear magnetization coupled to the feedback field of the radiofrequency (rf) coil.^6^ As a consequence, the spectral line shapes may be heavily distorted, which interferes with quantitative interpretation.^7^ In addition, uniform rf excitation over the entire spectral width is virtually impossible due to the large spectral line widths that are characteristic for static low‐temperature solid‐state NMR spectra. For these two main reasons, the quantitative evaluation of the line shapes in pulsed NMR spectra of supercooled samples is highly problematic (see the Supporting Information). However, high RD rates, $\lambda_{R}^{0}$ , also enable the use of an alternative detection method: spin noise detection.^8^, ^9^, ^10^, ^11^, ^12^, ^13^, ^14^ Observation of dynamic nuclear polarization (DNP)‐derived nuclear hyperpolarization through spin noise was achieved both after dissolution^15^ and more recently in situ.^16^ Because spin noise detection avoids the conversion of large longitudinal magnetization to transverse magnetization by rf pulses, there is no need to measure off‐resonance to avoid RD‐related line‐shape artifacts. As a further benefit, spin noise data are intrinsically free from any perturbations by rf‐pulse imperfections and interference.

Herein, we demonstrate spin noise detection as an alternative way of monitoring the very slow buildup of nuclear spin polarization of a sample in an ultralow temperature cryostat. The nuclear spin noise signal integral is related to the total longitudinal spin polarization by the current RD rate.

## 2 Theory

RD is a major source of complexity in NMR spectroscopy experiments on highly polarized samples. The origin of RD lies in the nature of the rf‐probe circuits used in NMR spectroscopy experiments.^6^ Any transverse magnetization induces a current in the receiver coil, which is normally detected as the free induction decay (FID). The same current also causes a magnetic feedback field (the RD field), which acts on the spins in turn, tilting the longitudinal magnetization away from the magnetic field axis, and thus, generating transverse magnetization. The rf‐field generated by this secondary transverse magnetization interferes with the original transverse signal in a complex way, depending on the phase shift of the feedback field.^17^, ^18^

Pronounced effects of RD are, for example, increased resonance line widths, line shapes that are, even for small flip angle spectra, no longer Lorentzian and may exhibit “wiggles” in the case of larger tipping angles.^7^ Although these effects are alleviated somewhat by fast transverse relaxation, they heavily interfere with quantitative interpretation.

For all NMR spectroscopy experiments with strong nuclear magnetization in resonant circuits, RD needs to be considered. The RD rate, $\lambda_{R}^{0}$ , is a measure for its impact, which is defined by Equation (1):^6^

(1) $$\lambda_{R}^{0}=\frac{1}{2}\eta \mu_{0}\gamma M_{z}^{0}Q$$

in which η is the filling factor,$\;\mu_{0}$ is the vacuum magnetic permeability, γ is the gyromagnetic ratio, $M_{z}^{0}$ is the thermal equilibrium magnetization, and Q is the quality factor of the rf circuit.

The high levels of magnetization, $M_{z}$ , that are achievable by hyperpolarization or brute‐force methods, together with resonance circuits of high quality factors, Q , and large filling factors,η , can cause huge RD rates. In some cases, these can counteract the original purpose of signal enhancement by circuit optimization and spin polarization.

To avoid more severe effects of RD on line shapes, small flip angle pulse spectra can be used,^7^ but even then the observed line shapes result from a complex interplay of *T*_1_, $T_{2}^{*}$ , and $\lambda_{R}^{0}\;$ , as well as RD.^7^, ^19^ RD can destroy the usually linear relationship between the observed signal amplitude and the amount of recovered magnetization.^20^ As a consequence the buildup curves observed on high polarization samples by pulsed NMR saturation recovery experiments may not deliver reliable information on the recovery of equilibrium magnetization, $M_{0}$ .

Spin noise spectra depend on the line width, $\lambda_{2}^{*}{=\left({\pi T}_{2}^{*}\right)}^{-1}$ , and on the equilibrium RD rate, $\lambda_{R}^{0}$ , and the actual RD rate,$\;\lambda_{R}\left(t\right)$ .^8^ The last one depends on the fraction of recovered magnetization, *K*(*t*)=*M*_z_(*t*)/*M*_0_, and thus, ${\;\lambda }_{R}\left(t\right)=\lambda_{R}^{0}K\left(t\right)$ . Notably, because we cannot clearly distinguish between homogeneous and inhomogeneous broadening herein, we use $\lambda_{2}=\lambda_{2}^{*}$ and $T_{2}=T_{2}^{*}$ herein and in the Supporting Information. Assuming perfect tuning (*ω*_LC_=*ω*_0_), the spin noise power spectrum is given by Equation (2):

(2) $$W_{\omega_{LC}=\omega_{0}}^{U}\left(\omega ,t\right)=\frac{2}{\pi }k_{B}TR_{P}\frac{{\left(\omega -\omega_{0}\right)}^{2}+\lambda_{2}\left(\lambda_{2}+\lambda_{R}^{0}\right)}{{\left(\lambda_{2}+\lambda_{R}^{0}K\left(t\right)\right)}^{2}+{\left(\omega -\omega_{0}\right)}^{2}}+W_{a}^{U}$$

in which *k*_B_ is the Boltzmann constant, *T* is the temperature of the coil and sample, *R*_P_ is the equivalent parallel resistance of the circuit, and $W_{a}^{U}$ is an additional noise source. The conditions of the experiments described herein are adequately described by Equation (2), so there is no need to use the refined theory recently introduced by Ferrand et al.,^21^ which covers more complex situations encountered under high‐resolution conditions. The influence of the temperature ratio between the sample and coil, *ϑ*, as well as of the tuning of the NMR receiver circuit have been described previously.^11^, ^22^, ^23^

After subtraction of the thermal circuit noise baseline [obtained from Eq. (2) with *M*_z_=0, and thus $\lambda_{R}^{0}=0$ ] from $W_{\omega_{LC}=\omega_{0}}^{U}\left(\omega ,t\right)$ , integration yields the theoretical spin noise signal integral [Eq. (3)].^11^, ^16^

(3) $$\lim_{D\to \infty }\int_{\omega_{0}-D}^{\omega_{0}+D}W_{\omega_{LC}=\omega_{0}}^{U}\left(\omega ,t\right)-W_{\omega_{LC}=\omega_{0},\;\;M=0}^{U}\left(\omega ,t\right)\;d\omega =2k_{B}TR_{P}\frac{\lambda_{R}^{0}\left(\lambda_{2}-2K\left(t\right)\lambda_{2}-{K\left(t\right)}^{2}\lambda_{R}^{0}\right)}{\left|\lambda_{2}+\lambda_{R}^{0}K\left(t\right)\right|}$$

in which *D* is used as an auxiliary variable for the integration limits of the symmetrical Lorentzian defined by Equation (2). This is the quantitative relationship between spin noise signal integral and fraction of recovered magnetization, *K*(*t*), which is discussed in more detail in the Supporting Information.

## 3 Results and Discussion

**Figure 1:**
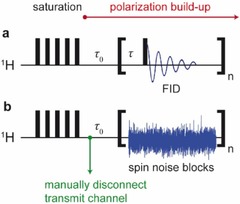
Pulse sequences for the acquisition of saturation recovery curves by using pulse excitation (a) and spin noise detected (b) spectra. For spin noise spectra, the transmit channel was manually disconnected following the saturation pulse train, and two 50 Ω terminators were connected to prevent signal pick‐up. The time required for the disconnection of the transmit channel and to start the monitoring experiments is $\tau_{0}$ .

Proton saturation recovery experiments conducted by using either conventional pulse excitation or the spin noise detection method introduced by McCoy and Ernst,^8^ shown in Figure 1 a and b, respectively, were performed at 2.5 T, at the resonant frequency of the rf circuit on a sample of a mixture of glycerol, water, and CuO nanoparticles (see the Experimental Section).

**Figure 2:**
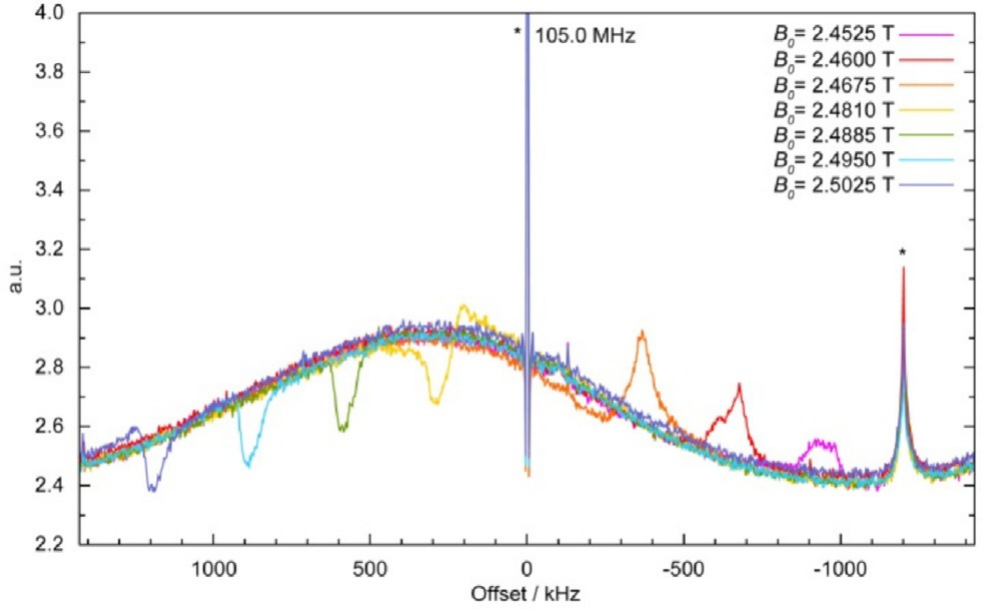
Experimental proton spin noise power spectra of a mixture of glycerol, water, and CuO nanoparticles (see the Experimental Section) at different field strengths (*B*_0_ according to the current set in the superconducting coil). The influence of the offset on the line shape can be observed. Artifacts are marked by asterisks. The central spike artifact at 105 MHz and external stray rf interference at −1200 kHz offset are independent of the magnetic field strength.

The magnetic field was adjusted such that a pure dip signal in the ^1^H spin noise spectra was obtained (see Figure 2). The “tuning” offset (here: offset of the Larmor frequency to the coil resonant frequency, as adjusted by the magnetic field strength) caused a dispersive contribution to the spin noise line shape.^8^

A fully negative absorptive line shape is not obtained at the Larmor frequency; this phenomenon cannot be explained by McCoy and Ernst's derivations^8^ and has been described as the spin noise tuning optimum (SNTO).^21^, ^22^ Adjusting for a negative purely absorptive spin noise peak (green trace in Figure 2) is an important prerequisite for performing the spin noise experiments shown herein.

**Figure 3:**
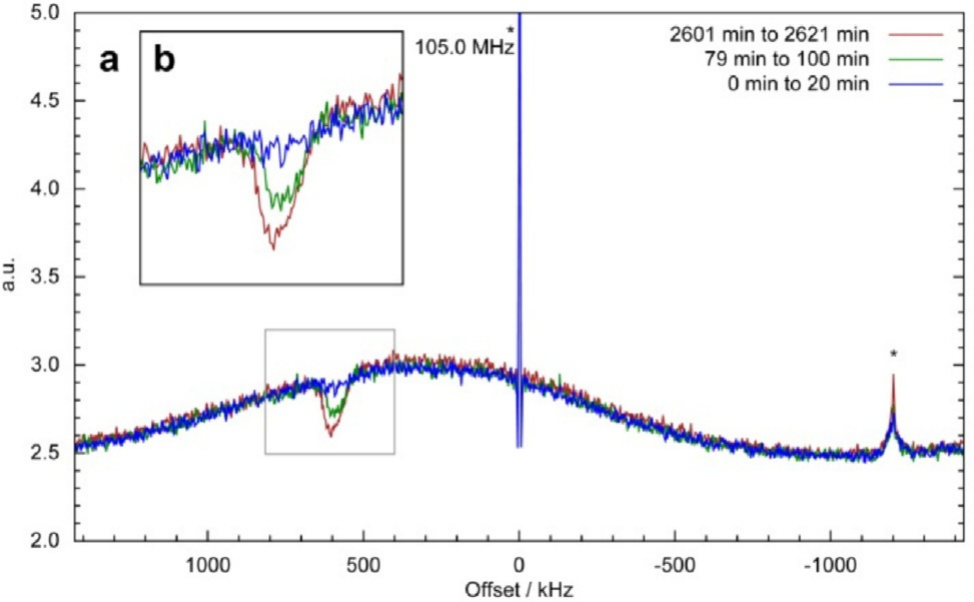
a) ^1^H spin noise power spectra recorded during buildup. The blue curve is the first acquired spectrum (recovery time: 10 min), the red curve is the last one (recovery time: 43 h 31 min), and one intermediate spectrum (recovery time: 1 h 30 min) is shown in green. The dip at +600 kHz is the growing spin noise signal. The Lorentzian‐shaped baseline is due to Nyquist noise.^16^, ^26^ Further peaks are artifacts: a central spike at 105 MHz and an external stray rf‐interference at an offset of −1200 kHz. b) An expanded region of the spectra indicated by the rectangle.

Three representative spectra acquired over the course of the spin noise saturation recovery experiment at 2.5 T are displayed in Figure 3. For comparison, additional experiments were performed by using the pulse method (Figure 1 a) at 2.5 (on‐resonance), 2.0, and 3.0 T (off‐resonance of the rf‐circuit). These data allow us to compare buildup rates determined by the standard saturation recovery method and to assess the suitability of the spin noise data for evaluation with respect to *T*_1_ field dependence. The saturation step was performed by using a train of hard pulses; its success was confirmed by the absence of a signal in the pulse spectra.

**Figure 4:**
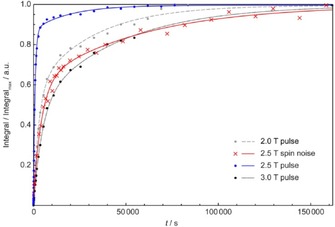
Saturation recovery curves at three different field strengths. Experiments with pulse spectra are marked by filled circles: in gray, at 2.0 T; in blue, at 2.5 T; and in black, at 3.0 T. The experimental data obtained from spin noise spectra at 2.5 T are indicated by red crosses. The fitted curves (see text) are plotted in the corresponding colors, by using a dotted line for 3.0 T and a dashed line for 2.0 T data.

The buildup curves obtained from all experiments have been plotted in Figure 4, each normalized to its maximum and superimposed for comparison.

The spin noise buildup data at 2.5 T (on‐resonance) measured by the spin noise acquisition scheme given in Figure 1 b are mostly located between the data points obtained at 2.0 and 3.0 T (off‐resonance), which are determined by pulsed NMR spectroscopy experiments by using the scheme shown in Figure 1 a. This conforms to the expected behavior because, generally, *T*_1_ increases with the strength of the magnetic field. The spin noise buildup data (red crosses in Figure 4) are closer to the two off‐resonance data sets (black and gray dots) than the pulse data points at 2.5 T (on‐resonance; blue dots in Figure 4). To analyze the differences between the data series in more detail, the experimental data were initially fitted to a monoexponential model. Similar to previous saturation–recovery pulse experiments under similar conditions,^4^ magnetization buildup was not monoexponential.

Because the mechanisms of relaxation in this complex system are still insufficiently explored, we focus on the quality of the experimental data with respect to systematic interference. Monoexponential target functions were insufficient for describing the experimental buildup data, so a two‐component model may represent the data more accurately. Notably, this does not imply that the biexponential model correctly represents the physical processes. The experimental recovery curves of the signal area, *A*(*t*), were fitted to Equation (4):

(4) $$A\left(t\right)=1-ae^{-t/T_{1a}}-be^{-t/T_{1b}}$$

in which *a* and *b* are the relative amplitude coefficients, and *T*_1a_ and *T*_1b_ are the relaxation time constants.

The parameters obtained by fitting Equation (4) to the experimental data in Figure 4 resulted in the curves shown in Figure 4, which are also summarized in Table 1.

**Table 1:** Parameters of curves fitted to the experimental saturation–recovery buildup data by using Equation (4). *R* ^2^>0.999 for all curves.

	$B_{0}$ [T]	a	$T_{1a}$ [s]	b	$T_{1b}$ [s]
pulse spectra^[a]^	2.0	0.421	3.11×10^4^	0.579	2.84×10^3^
pulse spectra^[a]^	2.5	0.124	1.89×10^4^	0.876	0.79×10^3^
spin noise power spectra	2.5	0.393	5.64×10^4^	0.567	4.34×10^3^
pulse spectra^[a]^	3.0	0.502	4.48×10^4^	0.498	4.63×10^3^

[a] Three‐parameter fit assuming *a*+*b*=1.

For experiments with pulse excitation, the additional condition *a*+*b*=1 was used in the fitting procedure. Because this assumption does not hold for spin noise based data, since even at full saturation a net spin noise signal should be observable,^8^, ^11^ *a* and *b* were treated as independent variables in the spin noise detected case.

The relaxation times determined by using on‐resonance spin noise detection are thus similar to those observed by pulse spectra off‐resonance, whereas on‐resonance strong RD causes systematic errors in pulse spectra. For both components, especially the more slowly recovering one, longer relaxation times are found by using spin noise detection. We see this as experimental evidence of the fact that spin noise observation does not interfere as much with the buildup process as pulsed observation experiments. The longitudinal relaxation times are expected to increase with increasing field strengths due to spin diffusion in the presence of the paramagnetic nanoparticles.^27^ Therefore, the relaxation times at 2.5 T are expected to be between those determined at 2.0 and 3.0 T. This is clearly obeyed for the spin noise data at 2.5 T of the faster component, whereas the pulsed experiment clearly violates this expectation.

No consistent trend can be derived for coefficients of the relaxation components. A detailed discussion of the aspects of quantitative interpretation of spin noise derived buildup curves, taking into account the intrinsically nonlinear behavior of spin noise integrals and potential systematic errors, can be found in the Supporting Information.

Notwithstanding the detailed analysis of the relaxation mechanisms in the presence of nanoparticles, we have compared the magnetic field dependence of the relaxation times to the simple power law dependence predicted spin diffusion models reported in Refs. 5, 27. As detailed in the Supporting Information, the on‐resonance spin noise relaxation times determined from the spin noise data on resonance agree with the expected monotonous field dependence much better than the values obtained by pulsed excitation. The field dependences of both longitudinal relaxation components, *T*_1*a*_ and *T*_1*b*_, in the sample investigated suggest rapid diffusion behavior, according to the definition of Blumberg.^27^

## 4 Conclusions

During ^1^H NMR spectroscopy experiments at millikelvin temperatures, high polarization caused strong RD, which interfered with the monitoring of polarization buildup by pulsed excitation spectra. We introduced the observation of nuclear spin noise as a viable alternative. With this approach, monitoring of the magnetization buildup was possible on‐resonance of the rf circuit. Buildup rates determined by means of spin noise detection on‐resonance were in the same range as those determined off‐resonance with pulse spectra. The application of the spin noise technique gave predictable responses to RD effects, and thus, allowed us to avoid using off‐resonance detection; this removed some experimental complexity and associated sources of uncertainty. In addition, even extremely broad line shapes could be detected reliably, since no limitation due to finite excitation bandwidth existed in spin noise detected NMR spectroscopy. As elaborated in the Supporting Information, potential systematic errors in relaxation times determined in this way were significantly below the statistically random errors usually encountered in pulsed relaxation experiments under conditions such as those used herein.

Preparation for spin noise detection was relatively straightforward to implement for slowly relaxing systems at extremely low temperatures: It required the spin noise signal to be located and the tuning of the coil or magnetic field strength to be adjusted for a purely absorptive spin noise line shape.

In particular, for monitoring the buildup of magnetization in very slowly relaxing systems, spin noise detection could improve the data quality. This method avoided any interference between polarization buildup and rf irradiation and could be applied continuously; thus making up for the inherently lower sensitivity.

## Experimental Section

All experimental data reported herein and in the Supporting Information were obtained from the protons in a sample of 23 μL total volume, which was prepared from 20 parts 2 m Na[1‐^13^C]acetate in H_2_O/glycerol=50:50 (v/v) and one part CuO nanoparticles (<50 nm).^5^ We performed the experiments at 17.5 mK on the millikelvin NMR spectrometer described in Refs. 4, 24, 25, which employed a ^3^He–^4^He dilution refrigerator and a superconducting magnet with a variable field strength of up to 15 T; we applied between 2.0 and 3.0 T. The NMR probe was tuned to a fixed frequency of 104.5 MHz, which corresponded to an approximate Larmor frequency of ^1^H at 2.5 T.

The pulse sequences used herein are shown in Figure 1. The magnetic field was kept constant during the entire duration of the saturation recovery experiments. The saturation sequence always consisted of 500 pulses of 2.5 μs at 18 W. For the conventional pulse experiments (Figure 1 a) used to monitor the recovery, 1 μs pulses at an rf power of 22 W were used. Due to the long relaxation times and frequent changes of the magnetic field, no exact pulse calibration was performed. Avoiding exact pulse angle calibration could be seen as an added advantage of spin noise based methods. Saturation recovery experiments with spin noise detection were performed analogously to the procedure introduced in Ref. 8 using the sequence in Figure 1 b for the monitoring of the extremely slow magnetization buildup pertinent here. To prevent pick‐up of rf signals through the transmitter cable and circuit acting as an antenna, the transmitter cable was manually disconnected from the probe after the saturation pulse train and two 50 Ω terminators were attached to avoid open connectors. Disconnecting the transmit channel was not a requirement for the experiment as such, but was a precaution required in the particular setup and environment. By using optimal spectrometer hardware and in an rf‐quiet environment (in particular, devoid of radio stations working in the respective frequency range), the connection could be left in place. The delay, *τ*_0_, required for this task and for starting the monitoring experiment was short (<1 min) relative to the relaxation times involved and the same for pulse and spin noise experiments, and thus, was negligible in the calculation of the time coordinates of the buildup curves. During the whole spin noise detected experiment, noise blocks of 1536 data points each were acquired with a spectral width of about 2.86 MHz, resulting in a duration of 0.26 ms per noise block. Each block was Fourier transformed and the power spectrum was calculated. A total of 16 k power spectra (corresponding to a period of 20 min of recovery time each) were then combined. To eliminate constant noise contributions from the spectrometer and environment, we subtracted a background noise power spectrum obtained by noise acquisition in an identical manner, except for the NMR Larmor frequency being set off‐resonance by changing the magnetic field to 3.00 T. (Notably, since that frequency was also off‐resonance with respect to the rf‐coil's resonance frequency, no spin noise signal was obtained.) A fifth‐order polynomial baseline correction was applied before final integration of the peaks. The resulting negative [due to the intrinsic properties of spin noise, see Eq. (2)] integral values were multiplied by −1 to enable direct comparison with conventionally determined buildup curves. For the spin noise detected spectra, the time coordinates in the buildup curves were calculated as the average of the start and end times of the noise block acquisition.

## Supporting information

As a service to our authors and readers, this journal provides supporting information supplied by the authors. Such materials are peer reviewed and may be re‐organized for online delivery, but are not copy‐edited or typeset. Technical support issues arising from supporting information (other than missing files) should be addressed to the authors.

Supplementary
