# Supplementary material for: Use of Nuclear Spin Noise Spectroscopy to Monitor Slow Magnetization Buildup at Millikelvin Temperatures
Source: Chemphyschem. 2016 Jul 22;17(19):3035–9. doi: 10.1002/cphc.201600323 (PMC5053266; doi:10.1002/cphc.201600323)
Supplement: Supplementary file 1 — Supplementary [file CPHC-17-3035-s001.pdf]

CHEMPHYSCHEM

Supporting Information

Use of Nuclear Spin Noise Spectroscopy to Monitor Slow Magnetization Buildup at Millikelvin Temperatures

Maria Theresia Pöschko,[a] David Peat,[b, c] John Owers-Bradley,[b] and Norbert Müller*[a, d]

cphc_201600323_sm_miscellaneous_information.pdf

1. Radiation damping effects on pulse spectra

On resonance, the signal is distorted (Figure S1) due to radiation damping (RD). Therefore, determinations of signal amplitudes and integrals are error-prone and unsuitable for the determination of build-up time constants.

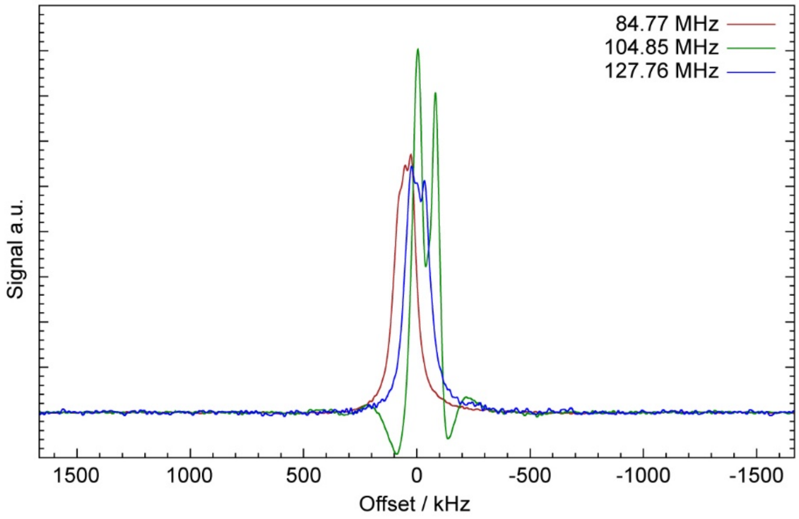

Figure S1: Overlaid ^{1}H NMR pulse spectra of the sample described in the Experimental Section of the main text showing signals on resonance (104.85 MHz, at 2.5 T, green) and off resonance (84.77 MHz at 2.0 T, 127.76 MHz at 3.0 T, red) of the rf-coil (tuned to 104.5 MHz, blue).

To avoid systematic errors in conventional (pulse excitation) build-up experiments at the high polarization levels encountered at extremely low temperatures, detection is usually carried out by detuning the rf-circuit or shifting the magnetic field away from the on resonance condition.

2. Field dependence of longitudinal relaxation times

Nuclear spin relaxation times are generally field dependent. In the presence of paramagnetic impurities like the nano-particles in the sample used here the magnetization recovery is mostly determined by the dynamics of spin diffusion.[S1] The appropriate spin diffusion model can be assessed by the field dependence of the T_1 relaxation times using the simple power model:[S1]

$$1/T_1 = c \cdot B_0{}^k \qquad \text{(S1)}$$

The exponent k is a measure for the prevailing relaxation mechanism.[S1] In Figure S1 the fits of Eq. S1 to our experimental data are shown. The fit parameters are summarized in Table S1.

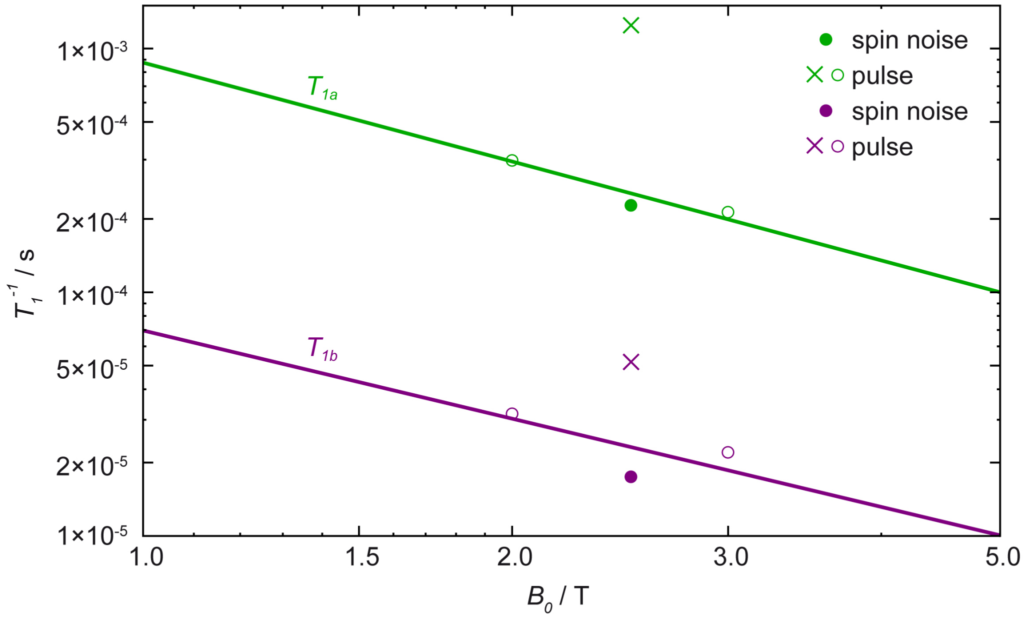

Figure S1: Experimental ^{1}H longitudinal reciprocal relaxation times of the two components a and b (see Eq. 4 in the main text) as functions of the magnetic field strength are plotted using logarithmic axes. Data pertaining to component a are displayed in green, for component b in purple. Crosses indicate data points obtained by on-resonance pulse excitation experiments. Filled circles represent the corresponding values obtained by spin noise detection. Off-resonance pulsed experimental data are indicated by empty circles. On-resonance spin noise data were fit together with the off-resonance pulse experiment data to the model in Eq. S1 as displayed by the straight lines.

Table S1: Results for the fit parameters for the field dependence of T_1 times shown in Fig. S1.

	c	k	R^2
a	0.00088	-1.35	0.996
b	0.00007	-1.20	0.975

As is evident from Fig. S1, the relaxation times determined on resonance by spin noise detection (filled circles, Figure S1) match to the monotonous power law much better than the ones determined by pulse excitation (crosses, Figure S1) on resonance. This is due to the systematic errors in the pulse experiments. (It does not mean that the true relaxation times are different for the two experiments.)

The values of the exponents k_a and k_b suggest that the rapid diffusion case[S1] is predominant in this sample.

3. Nuclear magnetic spin noise line shapes

Recently we introduced spin noise detection as a means to monitor the positive and negative polarization levels achieved by microwave irradiation in dynamic nuclear polarization (DNP) experiments.[S2] In this context we described the spin noise line shape following McCoy and Ernst:[S3]

$$W^U(\omega,t) = \frac{2}{\pi} k_B T R_P \frac{1+\lambda_R^0 a(\omega)}{[1+\lambda_R(t)a(\omega)]^2+[\lambda_R(t)d(\omega)+2Q(\omega-\omega_c)/\omega_c]^2} + W_a^U \tag{S2}$$

The spectral noise power density $W^U(\omega,t)$ thus depends on the temperature T, the equivalent parallel resistance of the circuit R_P, the radiation damping rate at thermal equilibrium λ_R^0, the current radiation damping rate $\lambda_R(t)$ and the resonance frequency of the coil ω_c as well as the additional noise sources W_a^U; k_B is the Boltzmann constant, $\lambda_2 = (\pi T_2)^{-1}$. Note that since we cannot clearly distinguish between homogeneous and inhomogeneous broadening here, we use the transverse relaxation rate $\lambda_2 = \lambda_2^*$ and $T_2 = T_2^*$ as in the main text. The radiation damping rate $\lambda_R(t)$ is zero after saturation and equal to λ_R^0 in thermal equilibrium. The enhancement factor K is used to describe the deviation from the thermal equilibrium, i.e. the relationship $\lambda_R(t) = K(t)\lambda_R^0$. The line shape is constructed from the absorptive $a(\omega)$ and dispersive $d(\omega)$ Lorentzian components

$$a(\omega) = \frac{\lambda_2}{{\lambda_2}^2+(\omega-\omega_0)^2} \qquad d(\omega) = \frac{\omega-\omega_0}{{\lambda_2}^2+(\omega-\omega_0)^2} \tag{S3}$$

Upon perfect tuning ($\omega_{LC} = \omega_0$) the equation reduces to Eq. 2 of the main text:

$$W_{\omega_{LC}=\omega_0}^U(\omega,t) = \frac{2}{\pi} k_B T R_P \frac{(\omega-\omega_0)^2+\lambda_2(\lambda_2+\lambda_R^0)}{(\lambda_2+\lambda_R(t))^2+(\omega-\omega_0)^2} + W_a^U \tag{S4}$$

In the absence of RD ($\lambda_R^0 = 0$ and $\lambda_R = 0$) no spin noise is detected, and the following equation describing the thermal Nyquist noise base line is obtained:

$$W_{\omega_{LC}=\omega_0,M=0}^U(\omega) = \frac{2}{\pi} k_B T R_P(\omega) \frac{1}{(1+2Q(\omega-\omega_c)/\omega_c)^2} + W_a^U \tag{S5}$$

Although spin noise line shapes strongly depend on the radiation damping rates, they are less susceptible to line shape distortions resulting from RD. Spin noise spectra may thus be perceived as corresponding to pulse spectra obtained at an infinitesimally small flip angle.

For the same conditions as for Eq. S4, the spin noise power signal integral calculates as (Eq. 3 of the main text)[S2,S4]

$$\lim_{D\to\infty} \int_{\omega_0-D}^{\omega_0+D} W_{\omega_{LC}=\omega_0}^U(\omega,t) - W_{\omega_{LC}=\omega_0,M=0}^U(\omega,t)\, d\omega = 2k_B T R_P \frac{\lambda_R^0(\lambda_2-2K(t)\lambda_2-K(t)^2\lambda_R^0)}{|\lambda_2+\lambda_R^0 K(t)|} \tag{S6}$$

Thus, the integrated spin noise power signal is non-linearly dependent on the enhancement factor $K(t)$.

4. Towards quantitative evaluation

An important consequence of Eq. S6 is that the signal shape and its sign change during a saturation recovery experiment: First, there will be a bump line shape and then there will be a dip line shape.[S3] The point where the spin noise signal is equal to the base line thermal Nyquist noise depends on λ_2 and λ_R^0 and is found at

$$K_0 = \frac{-\lambda_2 \pm \sqrt{{\lambda_2}^2+\lambda_2\lambda_R^0}}{\lambda_R^0} \tag{S7}$$

In Table S2, a number of thermal equilibrium radiation damping rates λ_R^0 is calculated to estimate where this point is positioned.

Table S2: Estimation of λ_R^0 from filling factor η and quality factor Q. (Temperature $T = 0.0175$ K, magnetic field strength $B_0 = 2.5$ T, and ^{1}H spin number density $n = 6.324 \times 10^{28}\ m^{-3}$)

λ_R^0 / rad s^{-1}		Q		
		70	90	110
η	0.05	76×10^3	98×10^3	120×10^3
	0.10	152×10^3	196×10^3	239×10^3
	0.15	228×10^3	293×10^3	359×10^3

In Figure S3a, the spin noise integral areas are drawn as a function of the enhancement factor K for the RD rates presented in Table S2 and assuming $\lambda_2/\pi = 110$ kHz. E.g., for $\lambda_r^0 = 196 \times 10^3$ rad/ s, there will be a bump in the spin noise power spectrum until an enhancement factor $K_0 = 0.45$ is reached (Figure S3a) and a dip will be observed.

In Figure S3b, the functions obtained for different radiation damping rates are displayed after normalizing to 1 at $K = 0$. Note that both slope and curvature vary with the assumed parameters.

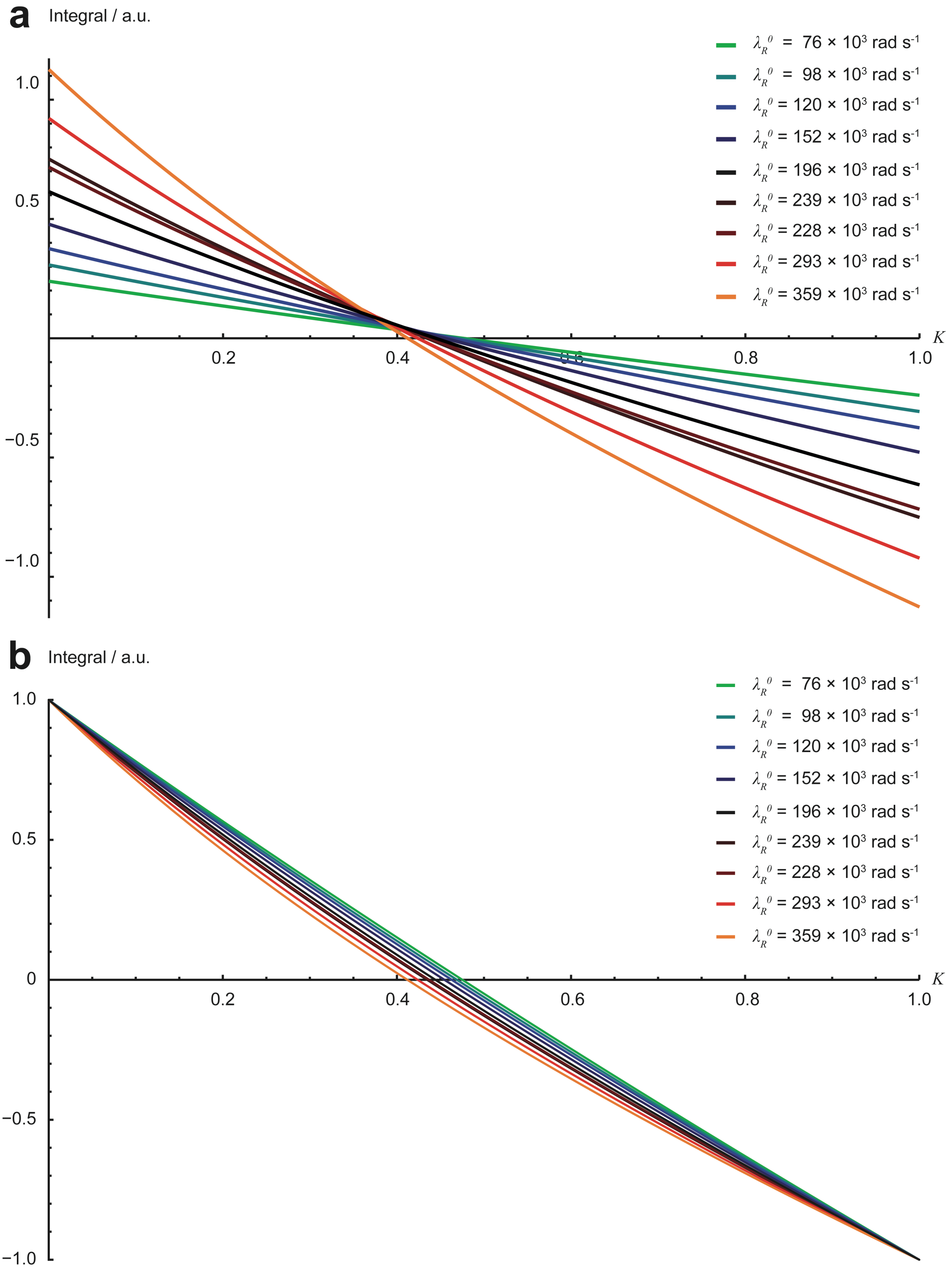

Figure S3: Dependence of the spin noise integrals on the enhancement factor K for a range of radiation damping rates. The black curve corresponds to the radiation damping rate $\lambda_r^0 = 196 \times 10^3$ rad/s as mentioned in the text and is found in the center of the bundle of curves. **(a)** The curves are not normalized: Larger radiation damping rates yield larger spin noise signal integrals. **(b)** Normalized curves corresponding to (a).

From Figure S4 the influence of the transverse relaxation rate (which had to be estimated in the experiments as described in the main text) can be estimated.

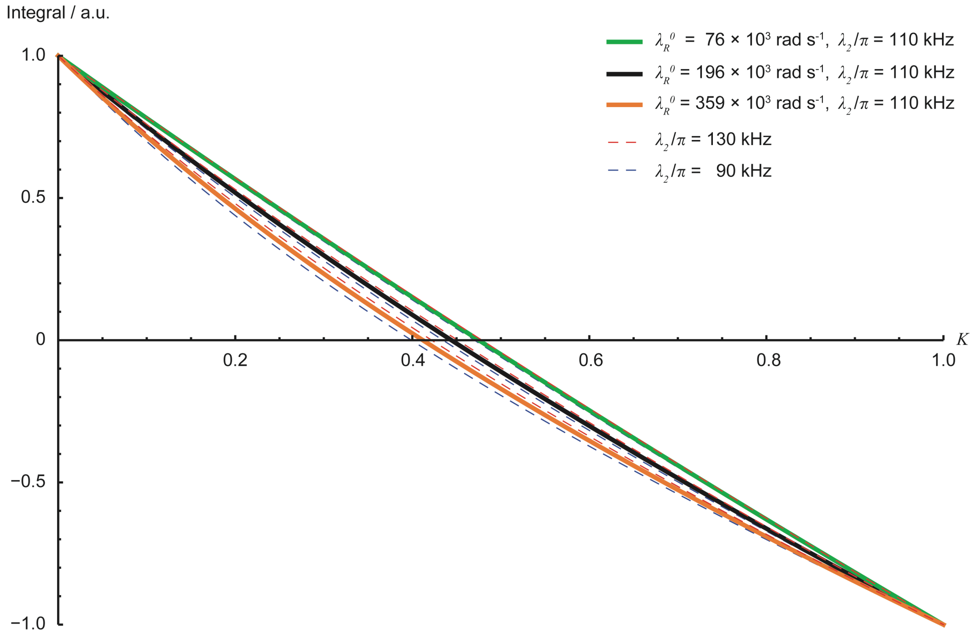

Figure S4: Illustration of the relative influence of three radiation damping rates (continuous lines) on the non-linear dependence of the spin noise signal integral on the deviation from equilibrium K and systematic errors caused by assuming $\lambda_2/\pi = \pm 20$ kHz (dashed lines).

If one normalizes the integrals between 0 and 1, the errors caused by λ_2 and λ_R^0 will influence the central part of the build-up curve most noticably. To estimate the systematic errors that may occur in quantitative evaluation of polarization from spin noise amplitude, recovery curves were calculated assuming a bi-exponential build-up using the same model as in the main text ($A(t) = 1 - ae^{-\frac{t}{T_{1a}}} - be^{-\frac{t}{T_{1b}}}$) in Figure S5, using the two outermost curves and the central black curve of Figure S4. The averages of the coefficients a and b and interpolated time constants T_{1a} and T_{1b} (from the pulse build-up experiments at 2.0 and 3.0 Tesla and the power model as in Equation S1), respectively, were used in this calculation:

$$a = 0.4615, \qquad T_{1a} = 38019\ \mathrm{s}, \qquad b = 0.5385, \qquad T_{1b} = 3717\ \mathrm{s}$$

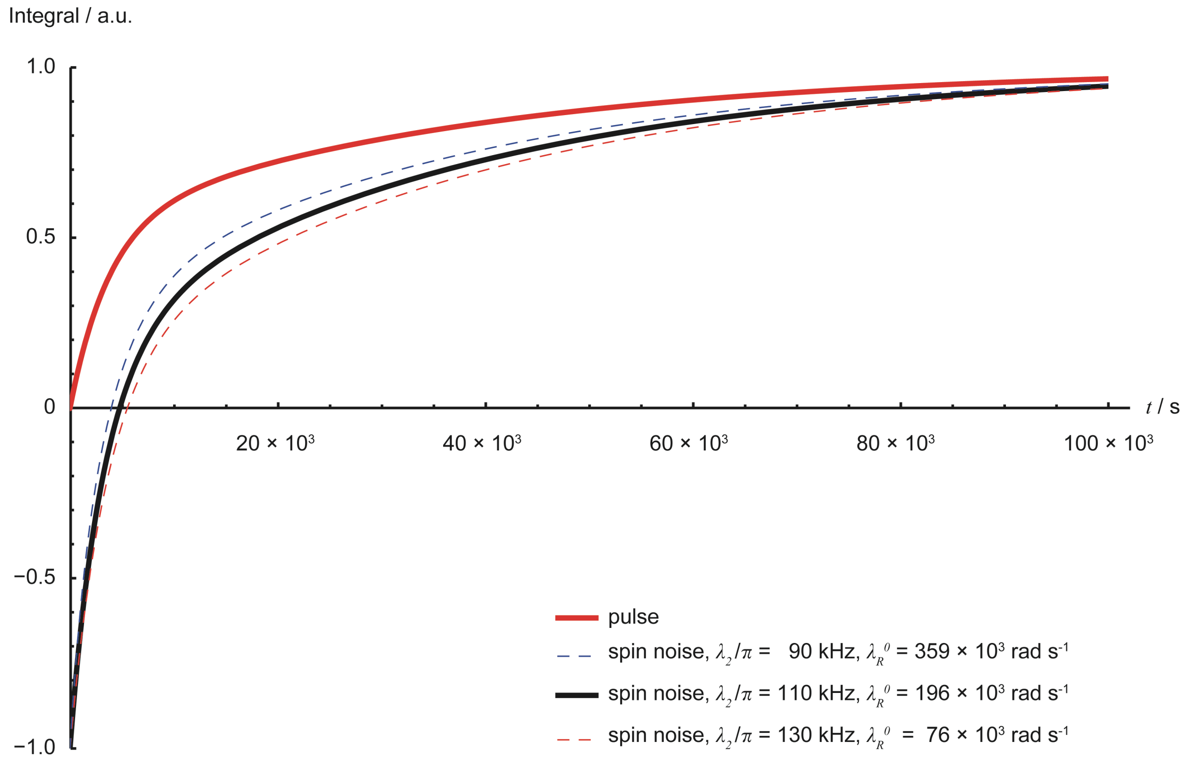

Figure S5: Computed ^{1}H signal build-up curves for bi-exponential recovery following saturation. The red line is the expected ideal build-up curve for a pulse experiment, neglecting any artifacts caused by RD and finite excitation bandwidth. The black trace corresponds to the same build-up observed by spin noise integrals. The blue and red dashed curves indicate the potential systematic error range based on maximum deviations in the assumed radiation damping and transverse relaxation rates.

The spin noise integral versus polarization curves in Fig. S4 are clearly non-linear, therefore direct quantification of the integrals to derive the polarization is not straightforward. However from Figures S3b, S4 and S5 we can deduce that these errors are small compared to the other experimental uncertainties present, in particular in the pulse experiment, due to the requirement for retuning or a field change for off-resonance measurements, as was used here.

5. Disagreement with experimentally observed build-up curves

The expected bump to dip transition (Figs. S3-S5) was not observed experimentally. In our experiments the first spin noise power spectrum recorded after saturation already shows a dip. Possible reasons for the missing of a positive signal could be:

(a) insufficient saturation which remains undetected in the pulse spectra due to too low signal to noise ratio.

(b) a very fast return to > 50% of the thermal equilibrium magnetization within the acquisition of the very first spin noise spectrum (20 min) – bump and dip components in the accumulated noise spectra cancel.

(c) inadequate approximations – the effect of the inhomogeneous broadening as well as the inhomogeneity of the B_1 field are neglected in equations S4 to S7

(d) inadequate estimates of λ_2 and λ_R^0 (λ_2 was estimated from line widths in off resonance pulse spectra).

For each of the cases it can be shown that the erroneous assumptions will have a major effect on the coefficients a and b but only minor changes of the relaxation times T_{1a} and T_{1b} will result. For example, if the starting point of the spin noise relaxation curve in Fig. S5 is erroneously shifted to the origin (by adjusting the first data point to 0 s) a fit according to Eq. 3 of the main text has been computed to assess the impact on the values for a, b, T_{1a} and T_{1b} as shown in Fig. S6.

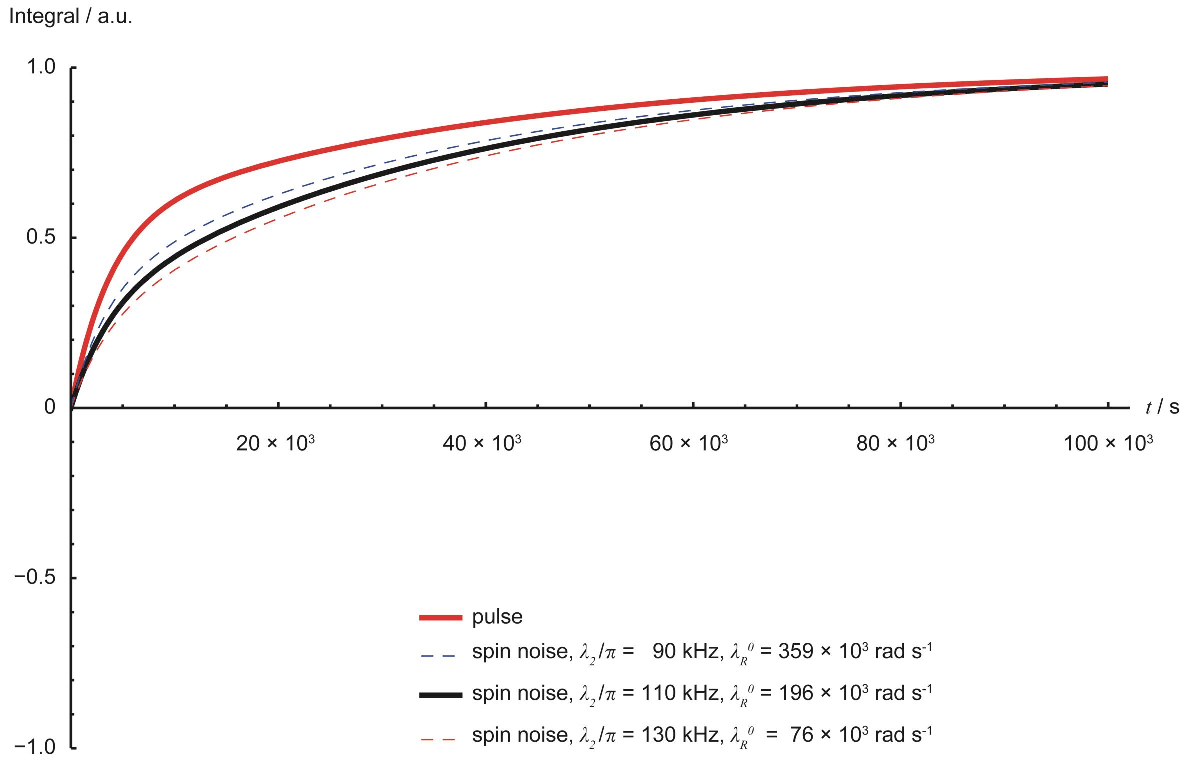

Figure S6: Simulated build-up curves analogous to Figure S5. Only the positive parts of the spin noise build up curves (corresponding to dip line shapes) are shown and shifted to start in the origin of the graph.

Using data points every 1 200 s from 0 to 100 000 s one can fit Eq. 3 of the main text to the simulated and shifted spin noise build up curves. The results of these fits are summarized in Table S3.

Table S3: Parameters of fitted curves to simulated, left shifted spin noise saturation-recovery data (Figure S6).

	a	T_{1a} / s	b	T_{1b} / s
Parameters for simulation	0.462	3.80×10^4	0.539	3.72×10^3
$\lambda_2 = 90$ kHz, $\lambda_R^0 = 359{\times}10^3$ rad/s	0.638	3.69×10^4	0.359	3.67×10^3
$\lambda_2 = 110$ kHz, $\lambda_R^0 = 196{\times}10^3$ rad/s	0.699	3.72×10^4	0.299	3.77×10^3
$\lambda_2 = 130$ kHz, $\lambda_R^0 = 76{\times}10^3$ rad/s	0.752	3.76×10^4	0.247	3.77×10^3

From the numbers in Table S3 one can derive deviations between the simulation parameters and the fitting results for the central black trace in Fig. S6 ($\lambda_2/\pi = 110$ kHz and $\lambda_R^0 = 196{\times}10^3$ rad/s):

$$a: +51\%, \qquad T_{1a}: -2\%, \qquad b: -44\%, \quad T_{1b}: +1\%$$

The strong influence on the coefficients a and b is inherent to the model, and the comparatively small impact on the relaxation times suggests that build-up curves determined by spin noise yield reliable relaxation times, even if the model used is not perfect. Note that the standard errors of the relaxation times in the fits to the experimental data (see Table S4) are nearly a magnitude higher than these potential systematic deviations.

Table S4: Fitting errors of the experimental spin noise saturation-recovery curve (2.5 T, black curve in Fig. 4 of the main text). R^2 was 0.9993.

	a	T_{1a} / s	b	T_{1b} / s
Parameter estimate	0.393	5.64×10^4	0.567	4.34×10^3
Standard error	0.022	0.48×10^4	0.027	0.45×10^3
Confidence interval	0.347<br>0.439	4.66×10^4<br>6.63×10^4	0.513<br>0.622	3.40×10^3<br>5.27×10^3

References

[S1] W.E. Blumberg, *Phys. Rev.* **1960,** *119*, 79–84.

[S2] M. T. Pöschko, B. Vuichoud, J. Milani, A. Bornet, M. Bechmann, G. Bodenhausen, S. Jannin, N. Müller, *ChemPhysChem* **2015,** *16,* 3859–3864.

[S3] M. A. McCoy, R. R. Ernst, *Chem. Phys. Lett.* **1989,** *159,* 587–593.

[S4] H. Desvaux, D. J. Y. Marion, G. Huber, P. Berthault, *Angew. Chem. Int. Ed.* **2009,** *48,* 4341–4343.

[S5] M. Nausner, J. Schlagnitweit, V. Smrečki, X. Yang, A. Jerschow, N. Müller, *J. Magn. Reson.* **2009,** *198,* 73–79.
